# Reduced Gray Matter Volume in Patients with Type 2 Diabetes Mellitus

**DOI:** 10.3389/fnagi.2017.00161

**Published:** 2017-05-22

**Authors:** Jia Liu, Taiyuan Liu, Wenhui Wang, Lun Ma, Xiaoyue Ma, Shaojie Shi, Qiyong Gong, Meiyun Wang

**Affiliations:** ^1^Department of Radiology, Henan Provincial People’s Hospital and the People’s Hospital of Zhengzhou UniversityZhengzhou, China; ^2^Department of Radiology, Union Hospital, Tongji Medical College, Huazhong University of Science and TechnologyWuhan, China; ^3^Huaxi MR Research Center, Department of Radiology, West China Hospital of Sichuan UniversityChengdu, China

**Keywords:** seed-based d mapping, voxel-based morphometry, meta-analysis, type 2 diabetes, gray matter

## Abstract

**Background and Purpose:** Previous studies of voxel-based morphometry (VBM) have found that patients with type 2 diabetes mellitus (T2DM) exhibit gray matter alterations, but these findings are inconsistent and have not been quantitatively reviewed. Therefore, the aim of this study was to conduct a quantitative meta-analysis of VBM studies of patients with T2DM.

**Materials and Methods:** The seed-based d mapping method was applied to quantitatively estimate the regional gray matter abnormalities in T2DM patients. We also used meta-regression to explore the effects of some demographics and clinical characteristics.

**Results:** Seven studies, with 8 datasets comprising 530 participants with T2DM and 549 non-T2DM controls, were included. The pooled and subgroup meta-analyses found that T2DM patients showed robustly reduced gray matter in the bilateral superior temporal gyrus, middle temporal gyrus, medial superior frontal gyrus, insula, median cingulate cortex, precuneus cortex and the left lentiform nucleus extending into the parahippocampus. The meta-regression also found that the percentage of female patients with T2DM was negatively associated with gray matter in the right superior temporal gyrus and illness duration was negatively associated with gray matter in the right middle temporal gyrus.

**Conclusion:** This meta-analysis indicates that T2DM patients have significantly and robustly reduced gray matter mainly in the cortical-striatal-limbic networks, which are associated with human cognition. Thereby implicating this finding in the pathophysiology of cognitive impairment in T2DM patients.

## Introduction

Type 2 diabetes mellitus is a common disease that impacts more than 360 million people worldwide, and its incidence may rise to 552 million cases by 2030 (Whiting et al., 2011). T2DM is increasingly being recognized as an important risk factor for cognitive impairment and dementia (Ding et al., 2010; Nooyens et al., 2010; Reijmer et al., 2010), which may eventually develop into Alzheimer disease. However, at present, the pathophysiological mechanisms underlying the cognitive impairment of T2DM patients remain poorly understood (Luchsinger, 2012). Brain imaging is an important tool for exploring the mechanisms linking T2DM and cognitive dysfunction. Structural MRI is one of the most common brain imaging modalities that can be used in cognitive research.

Studies using structural MRI have revealed reduced gray matter volume in the cortex-striatal-limbic regions, including the hippocampus, middle temporal cortex, superior temporal cortex, medial frontal gyrus and insula (Anan et al., 2011; Shimomura et al., 2011; Moran et al., 2013; Garcia-Casares et al., 2014; Zhang et al., 2014), in patients with T2DM. This reduction is thought to be related to the pathophysiology of cognitive impairment in T2DM. However, some of these studies used a seed-based ROI approach, in which researchers predefined several seeds with prior knowledge, which might have led to potentially biased results. VBM is an automated whole-brain-based analysis method that enables the unbiased investigation of gray matter differences between patients and controls (Ashburner and Friston, 2000). Therefore, it can overcome the limitations of the ROI method. Although studies using VBM have demonstrated abnormal gray matter volume alterations between patients with T2DM and non-T2DM controls, the results were inconsistent. For example, Gold et al. did not detect any significant gray matter differences between patients with T2DM and healthy controls (Gold et al., 2007), whereas other studies did observe such differences (Chen et al., 2012; Moran et al., 2013). Although most studies observed reduced gray matter volume in patients with T2DM, the results were also inconsistent. One study reported gray matter reductions in the right temporal lobe and precentral lobe (Chen et al., 2012), and another reported that the reductions were located mainly in the bilateral temporal lobe, precentral lobe and occipital lobe (Zhang et al., 2014). Many variables, such as the clinical and demographic characteristics of the patients, the sample size, the imaging devices and protocols used, may account for the observed inconsistencies. Thus, a meta-analysis is required to identify consistent results from VBM studies in patients with T2DM.

Seed-based d mapping is a statistical technique for meta-analyzing studies on differences in brain activity or structure which used neuroimaging techniques such as fMRI, VBM, DTI or PET. It is a new coordinate-based meta-analytic technique that has been applied to many diseases, including depression (Du et al., 2014; Zhao et al., 2014), post-traumatic stress disorder (Wang T. et al., 2016), migraine (Dai et al., 2015) and dementia (Zhong et al., 2014). The SDM method has been shown to be superior in some respects to the earlier methods, for example, activation likelihood estimation and multilevel kernel density analysis. For example, it can combine both positive and negative differences in the same map, thereby preventing a particular voxel from appearing to be significant in opposite directions (Radua et al., 2014). Additionally, SDM enables several complementary analyses, such as jack-knife, subgroup and meta-regression analyses, that can be used to assess the robustness and heterogeneity of the results (Radua et al., 2012). However, SDM has not been used in the meta-analysis of VBM studies comparing patients with T2DM and non-T2DM controls. Therefore, we set out to quantitatively review the published VBM studies on patients with T2DM and non-T2DM controls by using SDM to identify consistent regional gray matter abnormalities.

## Materials and Methods

### Inclusion of Studies

Our meta-analysis was conducted according to the guideline PRISMA (Moher et al., 2009). The studies were selected from the PubMed, Web of Science and Medline databases and were published between January 1947 and 2017. The keywords used were “diabetes” or “diabetes mellitus” plus “VBM,” “voxel-based,” “voxel-wise,” “morphometry,” or “VBM.” In addition, we manually checked the review articles and the reference lists of the identified articles. Articles were included if they met the following criteria: (1) they compared gray matter differences between patients with T2DM and non-T2DM controls at the whole-brain level, and (2) the gray matter differences between patients and controls were reported in a stereotactic space in three coordinates (*x*, *y*, *z*), including both MNI and Talairach coordinates. If some studies contained multiple independent patient samples, we included the coordinates as separate studies. Studies using ROI or seed voxel-based analysis were excluded. For studies lacking the Talairach or MNI coordinates, we contacted the authors in order to minimize the possibility of a biased sample set.

### Quality Assessment

A 13-point checklist was used to assess the quality of studies included. The checklist focused mainly on both the clinical and demographic aspects of individual study samples and the imaging-specific methodology. It was based on the previous meta-analytic studies (Shepherd et al., 2012; Du et al., 2014) and included the diagnostic procedures, the demographic and clinical characterization, the sample size, the MRI acquisition parameters, the analysis technique and the quality of the reported results (see Appendix, Supplementary Table S1). Although it was not designed as an assessment tool, the checklist can provide an objective indication of the rigor of individual studies. Each study was reviewed by at least two authors, and a completeness rating was independently determined. If ratings disagreements arose, the papers were discussed, after which a consensus score was obtained. The quality scores of each study are shown in **Table 1**.

**Table 1:** Demographic and clinical characteristics of the participants in the seven VBM studies (eight data sets) included in the meta-analysis.

Study	Patients with type 2 diabetes								Healthy controls				
				
	No (% female)	Mean age, year	Education year	Illness Duration, year	BMI kg/m2	HbAlc%	MMSE	Comorbidity (number of patients)	No (% female)	Mean age, year	Education year	MRI	Quality scores (out of 7)
Gold et al.	23 (52.2)	59.2	15.6	6	31.8	6.9	NA	Dyslipidemia or hypertension	23 (52.2)	59.9	15.9	1.5T	12.5
Chen et al.	16 (52.2)	61.2	NA	13.2	25.7	8.4	26	Hypertension (6)	16 (52.2)	59.8	NA	3T	11.5
Moran et al.	350 (40.0)	67.8	11.3	7	31.1	7.2	NA	Ischemic heart disease (82), TIA or stroke(37), hyperlipidemia (167)	363 (46.0)	72.1	10.9	1.5T	11
Garcia-Casares et al.	25 (32.0)	60.0	18.3	11.25	28.6	6.67	28.8	Hypertensive (11), dyslipidemia (11)	25 (44.0)	57.8	18.9	3T	12
Wang et al.	23 (30.4)	53.1	10.6	7	25.9	8.3	27.6	Hypertensive (10), dyslipidemia (8), lacunar infarcts(2)	23 (39.1)	53.9	10.7	3T	13
Zhang et al.^a^	25 (44.0)	52.2	12.9	6.44	24.29	7.39	27.84	NA	29 (58.6)	55.48	11.48	3T	12
Zhang et al.^b^	28 (71.4)	56.2	11.2	8.18	24.35	7.78	27.53	Mild cognitive impairment	29 (58.6)	55.48	11.48	3T	12
Ying et al.	40 (47.5)	60.5	10.0	8.90	24.4	7.7	28.3	Lacunar infarcts(9)	41 (68.3)	57.9	10.3	3T	12


*BMI, body mass index; MMSE, Mini Mental State Examination; TIA, transient ischemic attack; ^a^Subgroup of patients with type 2 diabetes but without mild cognitive impairment. ^b^Subgroup of patients with type 2 diabetes and comorbid mild cognitive impairment.*

### Voxel-Wise Meta-Analysis

Gray matter differences between patients with T2DM and non-T2DM controls were analyzed using SDM^1^, a voxel-based meta-analytic approach. First, we performed a pooled meta-analysis of the included studies. Then, a subgroup analysis, including data sets of patients with T2DM but without mild cognitive impairment, was performed. The SDM method has been described in detail elsewhere (Radua and Mataix-Cols, 2009; Radua et al., 2011, 2012), and we only describe it briefly here. First, the peak coordinates of the brain regions that were significantly different at the whole-brain level were selected. To avoid a potential bias toward liberally thresholded regions, we checked all the included studies to ensure that the same threshold was used throughout the brain. Second, we separately recreated a standard Talairach map of the differences in gray matter for each study by using a Gaussian kernel. The recreation of the peak coordinates was based on converting the peak *t-*value to Hedges effect size, and then applying a non-normalized Gaussian kernel to the voxels near the peak, which assigns higher values to the voxels closer to peaks. For null findings in the studies, the recreation was done with the same effect size, and all voxels in the effect size map were estimate to have a null effect size, which was the only difference. Similar to other effect sizes, the null effect size was also included in the random-effects meta-analytic models, which would modify the meta-analytic effect size. Third, the mean of the study maps were analyzed using a voxel-wise calculation to generate a mean map, and this calculation was weighted by the square root of the sample size of each study. Therefore, a study with a larger sample size would contribute more. Finally, we used standard randomization tests to determine statistical significance, hence creating null distributions from which *p* values were directly obtained. The default SDM kernel size and thresholds were used (full-width at half-maximum = 20 mm, voxel *p* = 0.005, peak height *Z* = 1, cluster extent = 10 voxels) (Radua et al., 2012). Additionally, we used a jack-knife sensitivity analysis to assess the robustness of the findings. For example, in the pooled meta-analysis, we repeatedly analyzed the data sets eight times, with one data set discarded each time. If a previously significant brain region remained significant in all or most of the combinations of the studies, it indicated that this finding is highly reproducible.

### Meta-Regression Analysis

Simple linear regression in the SDM can be used to examine the potential effects of the relevant sociodemographic and clinical variables presented in **Table 1** (Radua et al., 2012). The main output for each variable was a map of the regression slope. Based on the previous meta-analysis, to minimize the detection of spurious associations, we also decreased the probability threshold to 0.00005, required abnormalities to be detected both in the slope and in 1 of the extremes of the regressor, and discarded findings in regions other than those detected in the main analyses. Finally, we checked the regression plots to exclude fits that were driven by too few studies (Radua and Mataix-Cols, 2009).

## Results

### Studies Included in the Meta-Analyses

**FIGURE 1:**
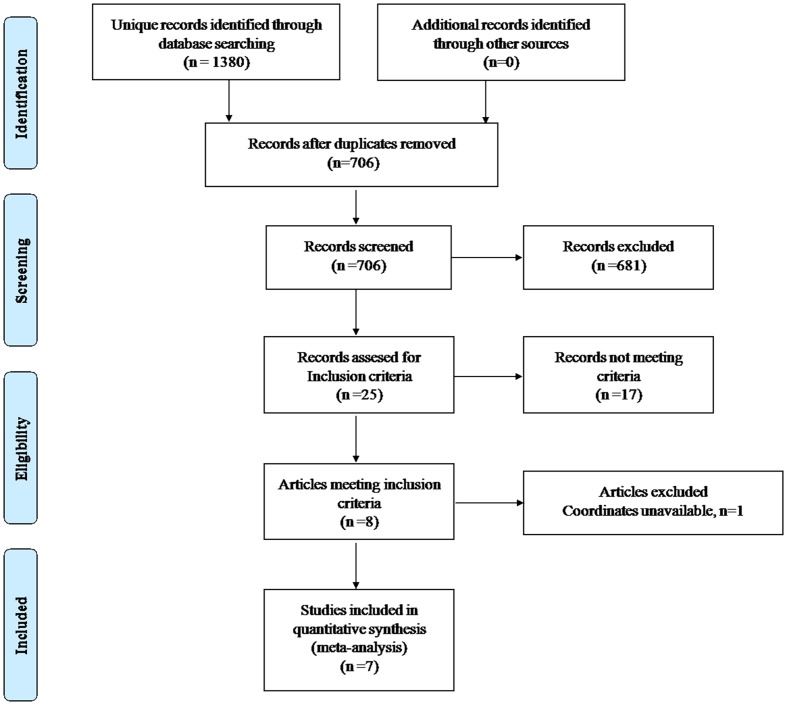
**Meta-analysis of VBM studies in patients with type 2 diabetes mellitus**.

The identification and attrition of the studies are shown in **Figure 1**. The search identified 1380 studies, and 7 VBM studies met the inclusion criteria. No additional articles were found in the reference lists of the selected studies. One of our included studies recruited patients with T2DM and with mild cognitive impairment. The analyses in this study were performed based on two different subgroups of patients with T2DM, who were then compared with the same healthy controls. Therefore, this study was treated as two unique studies, with each patient subgroup included independently in our meta-analysis. Finally, eight data sets were included in our study. Some basic information of the participants, such as clinical and demographic data, are shown in **Table 1**. There was no significant difference in age or sex between the T2DM and control groups in each study. In addition, no significant difference was found between these groups when considering the entire data set. Specifically, the mean age was 58.78 years in the T2DM group versus 59.04 years in the control group, and there were 229 (43.21%) women in the T2DM group versus 274 (49.91%) women in the control group.

### Pooled Meta-Analysis of All Studies

**FIGURE 2:**
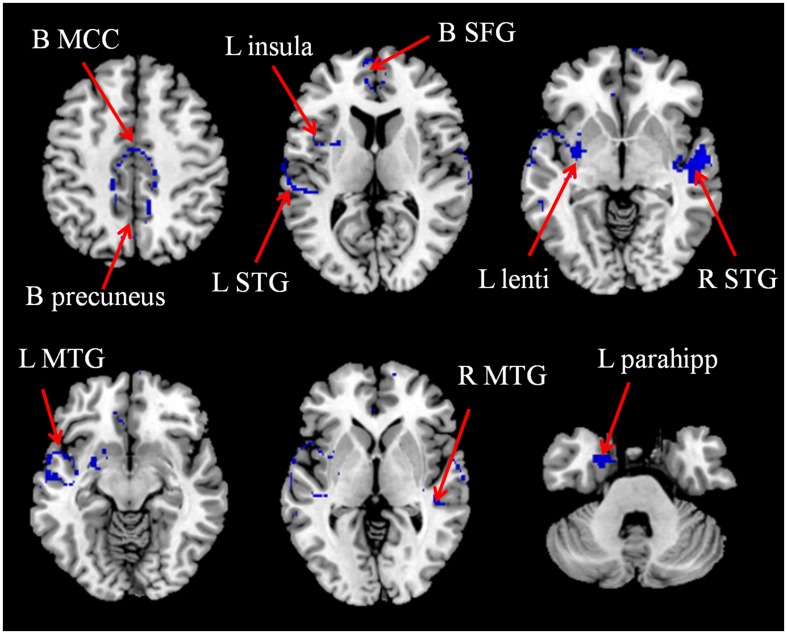
**The areas of decreased (blue) gray matter volumes in patients with T2DM compared with non-T2DM controls in the pooled meta-analysis.** B, bilateral; L, left; Lenti, lentiform nucleus; MCC, median cingulate gyri; MTG, middle temporal gyrus; parahipp, parahippocampus; R, right; SFG, superior frontal gyrus; STG, superior temporal gyrus.

In the pooled meta-analysis, gray matter volume in patients with T2DM was decreased, mainly in the bilateral STG, MTG, mSFG, insula, MCC, precuneus cortex and left lentiform nucleus extending into the parahippocampus, relative to the controls (**Table 2** and **Figure 2**).

**Table 2:** Regional differences in gray matter volume between patients with type 2 diabetes and healthy controls in the pooled meta-analysis (voxel-wise *p* < 0.005 and full-width at half-maximum 20 mm).

Brain Regions	Maximum			Clusters	
		
	MNI coordinates *x, y, z*	SDM value	*p*-value	No. voxel	Breakdown (no. of voxels)
**Diabetes < control**					
L superior temporal gyrus	-60,-26,10	-4.865	0.000010312	548	L superior temporal gyrus, BA21, 22, 28, 38, 41, 42, 48 (250)
					L insula, BA 48 (53)
					L middle temporal gyrus, BA20, 21, 22, 48 (78)
					L lenticular nucleus, putamen, BA 48 (41)
					L rolandic operculum, BA 22, 42, 48 (25)
					L parahippocampal gyrus, BA 28, 35, 36 (39)
					L amygdala, BA 34 (24)
					L striatum (15)
					L inferior frontal gyrus, orbital part, triangular part, opercular part, BA6, 11, 38, 48 (10)
					L supramarginal gyrus, BA 42, 48 (13)
R median cingulate/paracingulate gyri	2,-6,32	-4.668	0.000038207	460	R median cingulate/ paracingulate gyri, BA4, 23, 24 (89)
					L median cingulate/ paracingulate gyri, BA23, 24 (89)
					R precuneus, BA5, 23 (85)
					L precuneus, BA5, 7, 23 (75)
					L paracentral lobule, BA4 (35)
					R paracentral lobule, BA4 (20)
					L posterior cingulate gyrus, BA14, 26, 30 (29)
					R posterior cingulate gyrus, BA23, 26, 30 (25)
					L supplementary motor area, BA4, 6, 23 (21)
					R supplementary motor area, BA4, 6 (23)
R superior temporal gyrus	58,-10,-4	-5.224	∼0	271	R superior temporal gyrus, BA21, 22, 38, 42, 48 (171)
					R middle temporal gyrus, BA 21 (11)
					R insula, BA 48 (12)
					R post-central gyrus, BA22, 43, 48 (8) R rolandic operculum, BA22, 48 (7)
					R post-central gyrus, BA22, 43, 48 (12)
L superior frontal gyrus, medial	-8,56,8	-4.350	0.000253916	83	L superior frontal gyrus, medial, BA10, 32 (29)
					R superior frontal gyrus, medial, BA10 (8)
					L anterior cingulate/paracingulate gyri, BA10, 32 (33)
					R anterior cingulate/paracingulate gyri, BA10, 32 (13)
L middle temporal gyrus	-66,-32,-8	-4.398	0.001219988	25	L middle temporal gyrus, BA21, 22 (25)
R gyrus rectus	10,50,-18	-4.115	0.000847399	14	R gyrus rectus, BA 11 (14)
R supramarginal gyrus	62,-16,26	-4.184	0.000612080	11	R supramarginal gyrus, BA2, 43, 48 (15)
R superior frontal gyrus, orbital part	10,62,-18	-4.104	0.000903130	11	R superior frontal gyrus, orbital, BA 11 (8)


*BA, Brodmann area; L, Left; MNI, Montreal Neurological Institute; R, right; SDM, signed differential mapping.*

### Subgroup Meta-Analysis of Studies Including Patients without Mild Cognitive Impairment

We carried out a subgroup meta-analysis on the seven studies that included patients with T2DM but without mild cognitive impairment. In total, seven data sets that compared 702 patients without mild cognitive impairment and 520 healthy controls were analyzed (**Table 1**). The subgroup analysis also found gray matter volume decreases in patients relative to controls, and the decreases were located mainly in the bilateral STG, MTG, mSFG, insula, MCC, precuneus cortex and left lentiform nucleus extending into the parahippocampus (see Appendix, Supplementary Table S2).

### Reliability Analysis

As shown in **Table 3**, the whole-brain jack-knife sensitivity analysis of the pooled meta-analysis showed that the decreased gray matter volume in the bilateral STG, MTG, MCC, left insula and parahippocampus were highly replicable, as these findings were preserved throughout all eight combinations of the datasets. The reduced gray matter volume in the bilateral mSFG and precuneus, the left lentiform nucleus, the right insula were also significant in all but one combination of the data sets.

**Table 3:** Sensitivity analyses of VBM studies of gray matter in patients with type 2 diabetes in the pooled meta-analysis.

Discarded study	Decreased gray matter													
	
	L STG	L MTG	L insula	L puta	Lparahipp	L MCC	R MCC	R precu	L precu	R STG	R MTG	R insula	L SFG	R SFG
Gold et al.	Y^c^	Y	Y	Y	Y	Y	Y	Y	Y	Y	Y	Y	Y	Y
Chen et al.	Y	Y	Y	Y	Y	Y	Y	Y	Y	Y	Y	Y	Y	Y
Moran et al.	Y	Y	Y	N^d^	Y	Y	Y	N	N	Y	Y	N	N	N
Garcia-Casares et al.	Y	Y	Y	Y	Y	Y	Y	Y	Y	Y	Y	Y	Y	Y
Wang et al.	Y	Y	Y	Y	Y	Y	Y	Y	Y	Y	Y	Y	Y	Y
Zhang et al.^a^	Y	Y	Y	Y	Y	Y	Y	Y	Y	Y	Y	Y	Y	Y
Zhang et al.^b^	Y	Y	Y	Y	Y	Y	Y	Y	Y	Y	Y	Y	Y	Y
Ying et al.	Y	Y	Y	Y	Y	Y	Y	Y	Y	Y	Y	Y	Y	Y


*L, left; puta, putamen; MCC, median cingulate gyri; MTG, middle temporal gyrus; N, no; parahipp, parahippocampal gyrus; precu, precuneus; R, right; SFG, superior frontal gyrus; STG, superior temporal gyrus, Y, yes. ^a^Subgroup of patients with type 2 diabetes but without mild cognitive impairment. ^b^Subgroup of patients with type 2 diabetes and comorbid mild cognitive impairment. ^c^Remained significantly increased/decreased after exclusion of the study in the jack-knife analysis. ^d^No longer significantly increased/decreased after exclusion of the study in the jack-knife analysis.*

Whole-brain jack-knife sensitivity analyses of the subgroup meta-analysis of studies involving patients without mild cognitive impairment revealed that the decreased gray matter volume in the bilateral STG, left insula, SFG and right MTG were highly replicable, as these findings were preserved throughout all seven combinations of the datasets. The reduced gray matter volumes in the left MTG, precuneus, right insula and SFG were also significant in all but 1 combination of the data sets. The results in the bilateral MCC, left lentiform nucleus, parahippocampus and right precuneus remained significant in all but two combinations of the data sets.

### Meta-Regression

**FIGURE 3:**
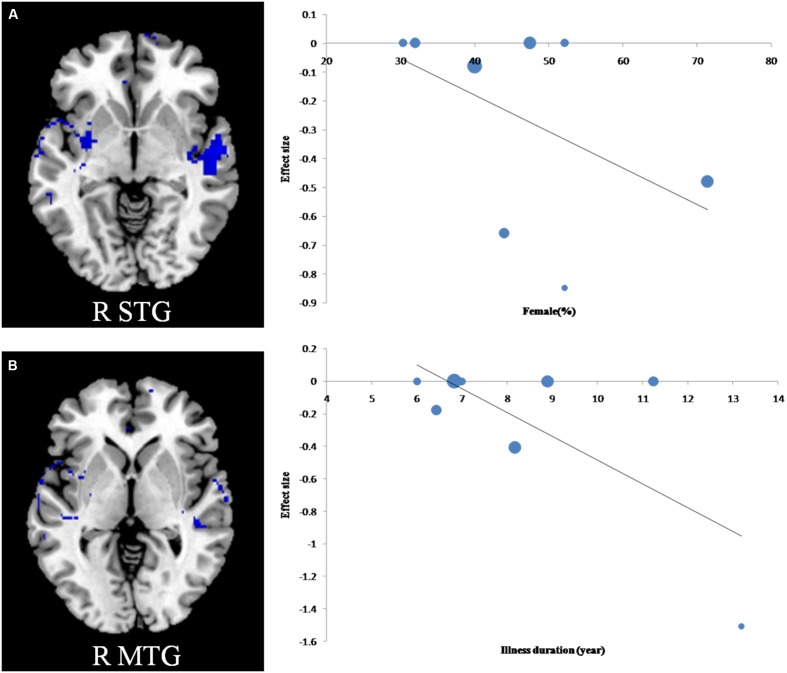
**Results of meta-regression analyses of studies of T2DM patients compared with non-T2DM controls.** **(A)** The percentage of female patients with T2DM was negatively associated with gray matter in the right superior temporal gyrus; **(B)** Illness duration was negatively associated with gray matter in the right middle temporal gyrus. In the graphs, the effect sizes needed to create this plot have been extracted from the peak of maximum slope significance, and each study is represented as a dot, whose size reflects samplesize. MTG, middle temporal gyrus; R, right.

The mean age, percentage of female patients with T2DM, illness duration, BMI and HbAlc% were explored by regression analyses in the patient group. These variables were available for all 530 participants in the 8 datasets. The percentage of female patients with T2DM was negatively associated with gray matter in the right STG (**Figure 3A**) and illness duration was negatively associated with gray matter volume in the right MTG (**Figure 3B**). However, these results should be interpreted with caution as they were driven by only three or four studies. The patients’ mean age, BMI and HbAlc% were not linearly associated with gray matter volume changes.

## Discussion

To our knowledge, this study is the first meta-analysis of VBM studies in patients with T2DM that examines how age and clinical characteristics affect the gray matter volumes. Both the pooled and subgroup meta-analyses identified reduced gray matter volume in the cortical-striatal-limbic networks. The results of our study may aid in the understanding of the underlying neurodegenerative process in T2DM. Additionally, this meta-analysis found that the percentage of female patients with T2DM was negatively associated with gray matter in the right STG and illness duration was negatively associated with gray matter volume in the right MTG.

Our meta-analysis found that only brain regions with reduced gray matter volume were detected and that brain regions with increased gray matter volume were not observed. One of the principal characteristics in T2DM is insulin resistance, which can reduce glucose metabolism and lead to increased plasma glucose in regional brain areas in T2DM patients (Baker et al., 2011). Hyperglycemia is considered the primary reason for diabetic complications in the brain because it may lead to various metabolic and molecular alterations and ultimately result in brain cell dysfunction or death (Tomlinson and Gardiner, 2008). Therefore, the resulting neuronal loss is observable as gross atrophy by MRI.

Most of the brain regions with reduced gray matter volume were located in the DMN, including the bilateral STG, MTG, MCC, mSFG, and precuneus. The DMN is known to participate in the core processes of human cognition, such as the integration of cognitive and emotional processing. Some researchers have labeled the DMN as the task-negative network (Fox et al., 2005) because it has been shown to be deactivated during external goal-oriented tasks, such as visual attention or cognitive working memory tasks. Many previous studies using functional connectivity have demonstrated that T2DM patients have a disrupted DMN. For example, using seed-based correlation analysis, one study found stronger correlations between posterior cingulate cortex and brain regions in the DMN (Musen et al., 2012), and another study found reduced functional connectivity between the hippocampus and some other brain regions in the DMN (Zhou et al., 2010). In addition, Wang et al. observed that T2DM patients exhibited a decreased amplitude of low-frequency fluctuations in the DMN (Wang et al., 2014). They also found that the altered amplitude of low-frequency fluctuations was associated with poor cognitive performance in patients with T2DM. Hoogenboom et al. (2014) investigated the relationship between structural and functional connectivity in T2DM patients and observed that fractional anisotropy of the cingulum bundle was correlated with functional connectivity between the brain regions of DMN (posterior cingulate and medial frontal gyrus). Thus, they suggested that therapies that improve one modality (i.e., structure or function) may affect the other as well. Therefore, we speculate that the decreased gray matter volume in the DMN results in dysfunction of functional connectivity or activity in the DMN in T2DM patients, which may eventually cause poor cognitive performance (Wang Y.F. et al., 2016). However, future studies are needed to confirm this hypothesis.

Gray matter decreases in the bilateral insula, the left putamen and parahippocampus were also detected in our study. These areas are important components of the limbic system, which supports various functions, including emotion, behavior, motivation and long-term memory (Morgane et al., 2005). Cui et al. (2014) studied the effects of glycemic variability at distinct time scales and found that subjects with greater glycemic variability both had less gray matter within the limbic system and exhibited worse cognitive performance. Many previous studies have also found that T2DM patients show abnormal spontaneous brain activity in these brain regions of the limbic system, based on assessments of the amplitude of low frequency fluctuations or regional homogeneity (Zhou et al., 2014; Peng et al., 2016). One study using voxel-based analysis found that patients with T2DM showed abnormal mean diffusivity in the left parahippocampus (Hsu et al., 2012). Although these studies have identified a connection between T2DM and the limbic system, because of the complex role of the limbic system in the brain, the precise association between T2DM and the limbic system still needs to be further explored.

Several limitations in our study should be highlighted. First, the small sample size may limit the power of our analyses. Second, the VBM method still has some limitations, although it has been well adapted for coordinate-based meta-analyses. Specifically, it may over represent group differences in brain regions with high anatomic variability, be biased toward finding highly circumscribed group differences and may not detect bran regions that are spatially more diverse (Bookstein, 2001; Tisserand et al., 2002). Finally, coordinate-based methods are based on summarized, rather than raw, statistical brain maps, which may result in less accurate results (Zhao et al., 2014).

## Conclusion

Our meta-analysis indicates that patients with T2DM have significantly and robustly reduced gray matter, mainly in the cortical-striatal-limbic networks. Our finding supports the notion that T2DM could lead to subtle diabetic brain structural changes, which may be correlated with cognitive impairment inT2DM patients. Longitudinal studies that investigate the dynamic brain structure changes of T2DM patients and the relationship between these alterations and cognition will help us better understand these results.

## Author Contributions

MW contributed to the conception of the study. JL, TL, WW, LM, XM, and SS contributed significantly to analysis and manuscript preparation. JL and TL performed the data analyses and wrote the manuscript. QG contributed to the interpretation and discussion of the results of the analysis.

## Conflict of Interest Statement

The authors declare that the research was conducted in the absence of any commercial or financial relationships that could be construed as a potential conflict of interest.

## Footnotes

1 http://www.sdmproject.com

## Abbreviations

BMI: body mass index
DMN: default mode network
MCC: median cingulate cortex
MNI: Montreal Neurological Institute
MRI: magnetic resonance imaging
mSFG: medial superior frontal gyrus
MTG: middle temporal gyrus
PRISMA: Preferred reporting items for systematic reviews and meta-analyses
ROI: region of interest
SDM: seed-based d mapping
STG: superior temporal gyrus
T2DM: type 2 diabetes mellitus
VBM: voxel-based morphometry
