# Supplementary material for: Reduced Gray Matter Volume in Patients with Type 2 Diabetes Mellitus
Source: Front Aging Neurosci. 2017 May 22;9:161. doi: 10.3389/fnagi.2017.00161 (PMC5439076; doi:10.3389/fnagi.2017.00161)
Supplement: Supplementary file 1 [file Table_1.doc]

**Table S1 Quality Assessment Checklist (When criteria were partially met, 0.5 points were assigned)**

| **Category 1: Participants** | Score (0/0.5/1) |
| --- | --- |
| 1. Patients were evaluated prospectively, specific diagnostic criteria were applied, and demographic data were reported.  2. Comparison participants were evaluated prospectively, psychiatric and medical illnesses were excluded.  3. Important variables (e.g., age, sex, illness duration, onset, medication status, BMI, HbA1c, intelligence quotient, i.e. IQ, handedness) were checked either by stratification or statistically.  4. Sample size per group > 10. | |
| **Category 2: Methods for image acquisition and analysis** | |
| 5. Whole brain analysis was automated with no a priori regional selection.  6. Magnet field strength > 1T.  7. MRI slice-thickness ≤ 3 mm and more than 1 slice was identified and traced.  8. Zero gap width.  9. Coordinates reported in a standard space.  10. The imaging technique used was clearly described so that it could be reproduced.  11. Measurements were clearly described so that they could be reproduced. | |
| **Category 3: Results and conclusions** | |
| 12. Statistical parameters for significant and important non-significant differences were provided.  13. Conclusions were consistent with the results obtained and the limitations were discussed. | |
| TOTAL /13 | |

**Table S2** Regional differences in grey matter volume between patients with type 2 diabetes and healthy controls in the subgroup meta-analysis (voxel-wise p < 0.005 and full-width at half-maximum 20 mm )

| Brain Regions | Maximum | | |  | Clusters | |
| --- | --- | --- | --- | --- | --- | --- |
|  | MNI coordinates  x, y, z | SDM value | p-value |  | No.  voxel | Breakdown  (no. of voxels) |
| **Diabetes<control** |  |  |  |  |  |  |
| L superior temporal gyrus | -48,-26,4 | -4.481 | 0.000041306 |  | 464 | L superior temporal gyrus, BA21, 22, 38, 41, 42, 48 (191) |
|  |  |  |  |  |  | L middle temporal gyrus, BA21, 22 (68) |
|  |  |  |  |  |  | L insula, BA48 (57) |
|  |  |  |  |  |  | L rolandic operculum, BA 42, 48 (34) |
|  |  |  |  |  |  | L lenticular nucleus, putamen, BA 48 (30) |
|  |  |  |  |  |  | L inferior frontal gyrus, orbital part, opercular part, triangular part, BA 6, 48 (22) |
|  |  |  |  |  |  | L parahippocampal gyrus, BA 28, 35, 36 (22)  L amygdala, BA 34, 36 (16) |
|  |  |  |  |  |  | L striatum (12)  L supramarginal gyrus, BA 42, 48 (12) |
| L median cingulate / paracingulate gyri | -16,-30,42 | -4.159 | 0.000322044 |  | 220 | L median cingulate / paracingulate gyri, BA23, 24 (51) |
|  |  |  |  |  |  | R median cingulate / paracingulate gyri, BA23, 24 (39)  R precuneus, BA5, 7, 23 (28)  L precuneus, BA5, 23 (33) |
|  |  |  |  |  |  | L paracentral lobule, BA 4 (24) |
|  |  |  |  |  |  | R paracentral lobule, BA 4 (15) |
|  |  |  |  |  |  | L supplementary motor area, BA 4, 6 (14)  R supplementary motor area, BA 4 (16) |
| R superior temporal gyrus | 58,-10,-4 | -4.788 | 0.000005186 |  | 150 | R superior temporal gyrus, BA 21, 22, 41, 48 (128) |
|  |  |  |  |  |  | R insula, BA 48 (12) |
|  |  |  |  |  |  | R middle temporal gyrus, BA 21 (5) |
|  |  |  |  |  |  | R rolandic operculum, BA 48 (4) |
|  |  |  |  |  |  | R lenticular nucleus, putamen, BA 48 (1) |
| L superior frontal gyrus, medial | -2,58,12 | -4.482 | 0.000041306 |  | 161 | L superior frontal gyrus, medial, BA 10,11, 12 (59)  R superior frontal gyrus, medial, BA 10 (21) |
|  |  |  |  |  |  | L anterior cingulate / paracingulate gyri, BA 10, 11, 32 (61)  R anterior cingulate / paracingulate gyri, BA 10, 11, 32 (20) |
| R median cingulate / paracingulate gyri | 12,-36,44 | 12,-36,44 | 0.000234306 |  | 111 | R median cingulate / paracingulate gyri, BA 23 (19)  R precuneus (34)  L precuneus, BA 23 (20)  L posterior cingulate gyrus, BA 23, 26, 30 (21)  R posterior cingulate gyrus, BA 23, 26, 30 (17) |
| R supramarginal gyrus | 62,-16,26 | -4.301 | 0.000146568 |  | 58 | R supramarginal gyrus, BA 2, 43, 48 (40)  R postcentral gyrus, BA 43, 48 (14)  R superior temporal gyrus, BA 48 (4) |
| R inusla | 36,-2,16 | -3.953 | 0.001140535 |  | 17 | R lenticular nucleus, putamen, BA 48 (13)  R insula, BA 48 (4) |
| L superior frontal gyrus, medial orbital | -4,68,-2 | -4.543 | 0.000452101 |  | 15 | L superior frontal gyrus, medial, medial orbital, BA 11 (3) |
|  |  |  |  |  |  | L gyrus rectus, BA 11 (12) |
| R inferior frontal gyrus, orbital part | 48,26,-10 | -4.233 | 0.001762927 |  | 19 | R inferior frontal gyrus, orbital part, triangular part, BA 45, 47 (17) |
|  |  |  |  |  |  | R insula, BA47 (2) |
| R rolandic operculum | 54,0,14 | -3.945 | 0.001193166 |  | 7 | R rolandic operculum, BA 48 (7) |

BA, Brodmann area; L, Left; MNI, Montreal Neurological Institute; R, right; SDM, signed differential mapping
